# *Phosphoserine Aminotransferase1* Is Part of the Phosphorylated Pathways for Serine Biosynthesis and Essential for Light and Sugar-Dependent Growth Promotion

**DOI:** 10.3389/fpls.2018.01712

**Published:** 2018-11-20

**Authors:** Sabine Wulfert, Stephan Krueger

**Affiliations:** Biocenter – Botanical Institute II, University of Cologne, Cologne, Germany

**Keywords:** serine biosynthesis, primary metabolism, photorespiration, amino acid metabolism, growth promotion, *Arabidopsis thaliana*

## Abstract

The phosphorylated pathway of serine biosynthesis represents an important pathway in plants. The pathway consist of three reactions catalyzed by the phosphoglycerate dehydrogenase, the phosphoserine aminotransferase and the phosphoserine phosphatase, and the genes encoding for all enzymes of the pathway have been identified. Previously, the importance of the phosphoglycerate dehydrogenase and phosphoserine phosphatase for plant metabolism and development has been shown, but due to the lack of T-DNA insertion mutants, a physiological characterization of the phosphoserine aminotransferase is still missing. Hence, we generated silencing lines specifically down-regulated in the expression of the major *PSAT1* gene. The morphological characterization of the obtained *PSAT1*-silenced lines revealed a strong inhibition of shoot and root growth. In addition, these lines are hypersensitive to the inhibition of the photorespiratory serine biosynthesis, when growing the plants at elevated CO_2_. Metabolic analysis of *PSAT1*-silenced lines, showed a strong accumulation of certain amino acids, most likely due to an enhanced ammonium assimilation. Furthermore, phenotypic analysis under low and high-light conditions and in the presence of sucrose revealed, that the phosphorylated pathway of serine biosynthesis is essential for light and sugar-dependent growth promotion in plants.

## Introduction

Serine is an important intermediate in various metabolic pathways in plant metabolism, including photorespiration, the biosynthesis of phospholipids, the one-carbon metabolism and the synthesis of amino acids, such as glycine, methionine, cysteine, and tryptophan (Vance and Steenbergen, 2005; Rebeille et al., 2007; Krueger et al., 2009; Tzin and Galili, 2010). In yeast, serine is synthesized by two pathways, the phosphorylated pathway of serine biosynthesis (PPSB), and the gluconeogenic pathway (Pizer, 1963; Snell, 1984; Melcher and Entian, 1992; Achouri et al., 1997; Dey et al., 2005). In most other organisms, such as mammals, algae and bacteria, serine is mainly synthesized by the PPSB (Pizer, 1963; Snell, 1984; Melcher and Entian, 1992; Achouri et al., 1997; Dey et al., 2005).

In plants, the situation is more complicated since serine is synthesized by three different pathways, the photorespiration, the glycerate pathway and the PPSB (Ros et al., 2014).

The photorespiratory pathway starts with the synthesis of 2-phosphoglycolate by the oxygenase reaction of ribulose-1,5-bisphosphate carboxylase/oxygenase. Via multiple reactions localized in chloroplasts and peroxisomes 2-phosphoglycolate is converted to glycine and subsequently to serine. More than 70% of the serine synthesized by the photorespiration is recycled by the serine glyoxylate aminotransferase reaction to feed back the carbon into the Calvin cycle and only 30% is used to satisfy the demand of serine for other cellular processes (Busch et al., 2018).

**FIGURE 1:**
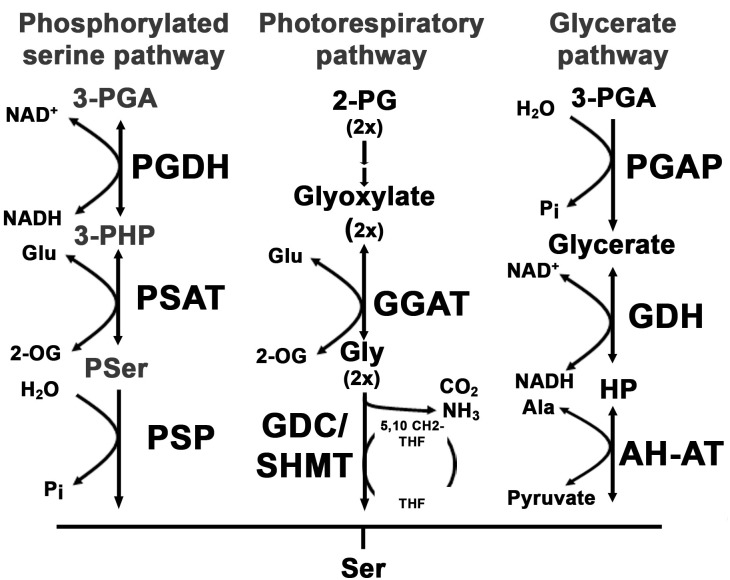
Overview of serine biosynthesis pathways in plants. In plants, serine can be synthesized via three pathways: the phosphorylated serine biosynthesis pathway, the photorespiratory pathway and the glycerate pathway. The first reaction within the phosphorylated serine biosynthesis pathway is catalyzed by the phosphoglycerate dehydrogenase (PGDH) enzyme. The PGDH enzyme oxidizes 3-phosphoglycerate (3-PGA) to 3-phosphohydroxypyruvate (3-PHP) by reducing NAD to NADH(H). In the next step the amino group of glutamate is transferred to 3-PHP by the phosphoserine aminotransferase (PSAT) enzyme, yielding phosphoserine (Pser) and 2-oxoglutarate (2-OG). Finally, Pser is dephosphorylated to serine by the phosphoserine phosphatase (PSP). The photorespiratory pathway starts with the conversion of 2-phosphoglycolate (2-PG) to glyoxylate. The glyoxylate glutamate aminotransferase (GGAT) transfers the amino group from glutamate to glyoxylate yielding glycine and 2-OG. Glycine is further decarboxylated by the glycine decarboxylase complex (GDC) releasing CO_2_ and NH_3_ and yielding one molecule of methylated tetrahydrofolate (5,10-CH_2_-THF). The methyl group of 5,10-CH_2_-THF is transferred to an additional glycine to form serine catalyzed by the serine hydroxymethyltransferase (SHMT). The third serine biosynthesis pathway catalyzes serine from 3-PGA. 3-PGA is dephosphorylated to glycerate by the 3-PGA phosphatase (PGAP). Glycerate is further oxidized to hydroxypyruvate (HP) and finally converted to serine by transferring the amino group from alanine. The last reaction is catalyzed by the alanine – HP aminotransferase (AH-AT).

The biosynthesis of serine by the glycerate pathway represents the reverse sequence of reactions of the photorespiration, from 3-phosphoglycerate to serine (Figure 1; Kleczkowski and Givan, 1988). Although, the activity of all the enzymes involved in the glycerate pathway could be measured in plant extracts, the physiological relevance of the pathway is still not confirmed (Ros et al., 2014).

Within the PPSB serine is synthesized from 3-phosphoglycerate and the pathway consists of three sequential reactions that are catalyzed by 3-phosphoglycerate dehydrogenase (PGDH), 3-phosphoserine aminotransferase (PSAT), and 3-phosphoserine phosphatase (PSP) enzymes (Ho and Saito, 2001). All enzymes of the PPSB are localized in plastids and most of the genes are dominantly expressed in heterotrophic tissue, except *PGDH3*, which is expressed only in leaves and *PSP*, which is expressed in both leaf and root tissue (Benstein et al., 2013).

The coexistence of three serine biosynthetic pathways in plants complicates the understanding of serine homeostasis in plants.

Photorespiratory serine biosynthesis is intimately associated with photosynthesis and therefore restricted to photosynthetic tissue. In the absence of photorespiration, serine is most likely synthesized either by the glycerate pathway or the PPSB.

The PPSB enzymes are encoded in plants by small gene families. The Arabidopsis genome contains three genes for the PGDH (*At4g34200*, *PGDH1*; *At1g17745*, *PGDH2*; *At3g19480*, *PGDH3*), two genes for the PSAT (*At4g35630*, *PSAT1*, and *At2g17630*, *PSAT2*) and one gene for the PSP (*At1g18640*). Deficiency in either the *PGDH1*, or the *PSP* gene causes embryo lethality and male sterility and plants with reduced activity of PGDH1 or PSP showed a strong inhibition of shoot and primary root growth (Benstein et al., 2013; Cascales-Minana et al., 2013). In addition, plants simultaneously inhibited in photorespiration and PPSB activity are completely arrested in growth (Benstein et al., 2013), indicating that these two pathways are of major importance for serine homeostasis whereby the glycerate pathway seems to play only a minor role in plants. It has been proposed that the PPSB is of increased relevance during the dark (Cascales-Minana et al., 2013), but there is substantial evidence that the PPSB is also essential in the light (Ho et al., 1998).

In contrast to the strong phenotype of *PGDH1* mutant plants, mutation in either *PGDH2* or *PGDH3* does not result in any obvious phenotype (Benstein et al., 2013; Toujani et al., 2013). Therefore, the role of these two genes in serine biosynthesis via the PPSB is still not known. In addition, due to the lack of T-DNA insertion mutants, it is still unclear whether the two genes, annotated as *PSAT*, encode for functional PSAT enzymes and whether these enzymes contribute to serine biosynthesis via the PPSB. The PSAT belongs to the group of aminotransferase enzymes. Aminotransferases catalyze the reversible transfer of amino groups from amino acids to oxoacids and are involved in many different reactions in plants (Mehta et al., 1993). The *in vitro* analysis of several aminotransferases revealed that some are highly substrate specific (Hirayama et al., 1998), whereas others are rather unspecific (Zhang et al., 2013). A prominent example for the broad substrate range of some aminotransferases is the serine: glyoxylate aminotransferase (AGT1) (Zhang et al., 2013). This enzyme catalyzes the transfer of amino groups from alanine, serine, glycine and asparagine to several different oxoacids *in vitro*. Overexpression of the AGT1 enzyme in plants alters significantly the content of serine, alanine and asparagine (Modde et al., 2017), indicating that this enzyme also catalyzes several reactions *in vivo*. Thus, the analysis of the *in vivo* function of aminotransferase enzymes is essential to understand their function in plants.

**FIGURE 2:**
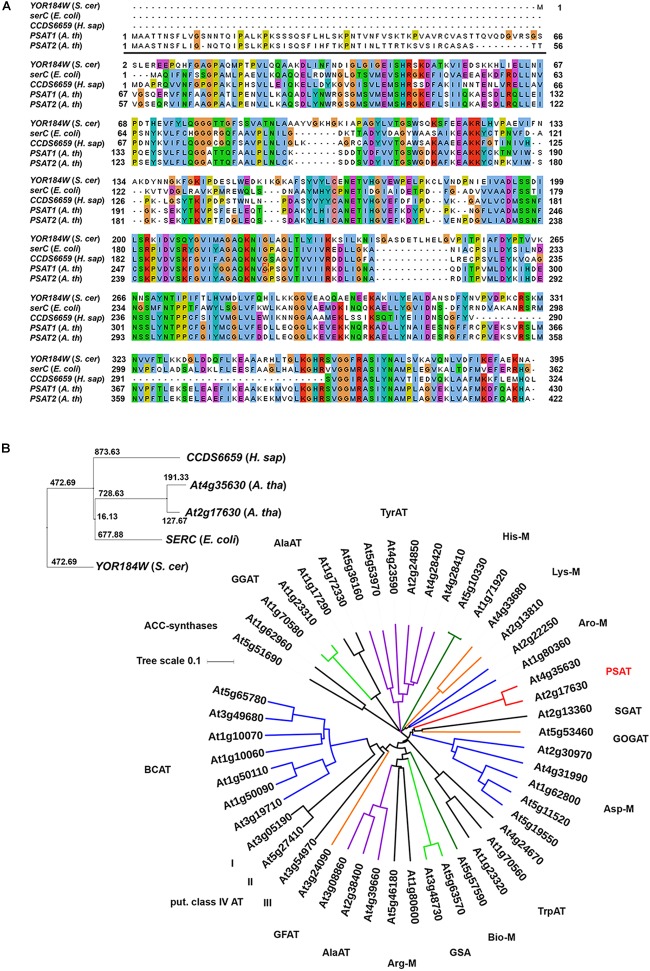
Sequence comparison of PSAT proteins. Sequence comparison of PSAT proteins of *Escherichia coli*, *Saccharomyces cerevisiae, Homo sapiens*, and *Arabidopsis thaliana* **(A,B)**. Phylogenetic tree of proteins annotated as aminotransferase enzymes in the ARAMEMNON database (Schwacke et al., 2003), release 8.1 **(B)**. ACC, amino-cyclopropane-1-carboxylate)-synthases; GGAT, glutamate:glyoxylate aminotransferase; AlaAT, alanine aminotransferase; TyrAT, tyrosine aminotransferase;, His-M, aminotransferases involved in histidine metabolism; Lys-M, aminotransferases involved in lysine metabolism; Aro-M, aminotransferases involved in biosynthesis of aromatic amino acids; PSAT, phosphoserine aminotransferase; SGAT, serine:glyoxylate aminotransferase; GOGAT, glutamate:oxoglutarate aminotransferase; Asp-M, aminotransferases involved in aspartate metabolism; TrpAT, tryptophan aminotransferase; Bio-M, aminotransferases involved in biotin metabolism; GSA, aminotransferases involved in chlorophyll biosynthesis; Arg-M, aminotransferases involved in arginine metabolism; GFAT, glutamate:fructose aminotransferase; BCAT, branched chain aminotransferases.

Database searches revealed that the two putative PSAT enzymes form a clearly isolated clade within the large group of annotated plant aminotransferases (Figure 2). In addition, these enzymes showed no homology to other proteins^1^, indicating that these two enzymes are the only PSAT proteins in Arabidopsis. To study the function of the putative *PSAT* genes in Arabidopsis, we analyzed their expression pattern, generated silencing lines to down-regulate the major expressed *PSAT1* gene and functionally characterized these lines under various growth conditions.

## Results

### Identification and Expression Patterns of *PSAT* Genes in *Arabidopsis thaliana*

Two genes, *PSAT1* (At4g35630) and *PSAT2* (At2g17630) with a high similarity to the *SERC* gene from *Escherichia coli*, the *CCDS6659* gene from *Homo sapiens* and the *YOR184W* gene from *Saccharomyces cerevisiae* were identified in the *Arabidopsis thaliana* genome (Figure 2). Homologous *PSAT* genes were further identified in the genome of all sequenced green plants present in the Phytozome 12 database^2^. The number of *PSAT* isoforms present in the different plant genomes is rather diverse. Most of the plants contain two *PSAT* genes, whereas others contain only one *PSAT* isoform or up to five (Supplementary Figure 1). In Arabidopsis, the two PSAT isoforms contain a putative target peptide for localization into plastids (Figure 2A). The consensus prediction for plastid localization of both PSAT proteins is with a score of 26.2 for PSAT1 and 27.3 for PSAT2, fairly high, indicating that both proteins are plastid localized.

**FIGURE 3:**
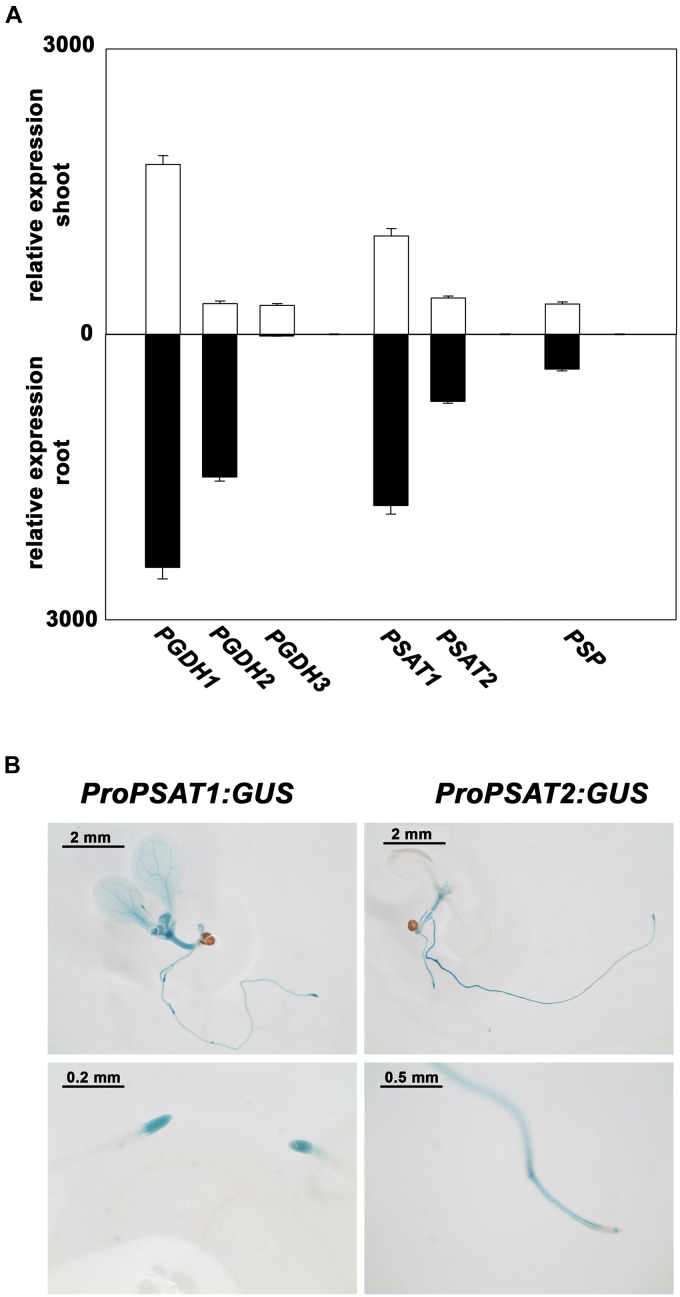
Expression pattern of PPSB genes in *Arabidopsis thaliana.* Expression level of PPSB genes in shoot and root tissue of Arabidopsis plants obtained from publicly available resources (http://bar.utoronto.ca/) **(A)**. For analysis of the spatial distribution of *PSAT1* and *PSAT2* expression, plants expressing the *GUS* gene under the control of either the *PSAT1* or *PSAT2* promoter were analyzed **(B)**.

*PSAT1* is the most expressed isoform and the expression of both genes is higher in root than in shoot tissue (Figure 3A). In order to study the tissue specific expression pattern of *PSAT* genes in more detail, *Arabidopsis* plants expressing the β-glucuronidase (*GUS*) reporter gene under the control of either *PSAT1* or *PSAT2* promoter were analyzed (Figure 3B). In 10 days old plants the activity of both *PSAT* gene promoters is high in the shoot apical meristem (SAM), the root apical meristem (RAM), in the vasculature of the hypocotyl and in the vasculature of roots, whereas the *PSAT1* promoter is additionally active in the vasculature tissue of cotyledons and young leaves. In general, the expression pattern of both *PSAT* genes resembles rather the expression pattern of the *PGDH1* and *PGDH2* gene, with a higher expression in root than in shoot tissue, than the expression of the *PSP* gene. The gene encoding for the PSP is much weaker expressed than the other genes and the expression is similar in root and shoot tissue.

### Reduction of *PSAT1* Gene Expression Severely Impairs Plant Growth

The *PSAT1* gene is the most expressed isoform in *Arabidopsis thaliana* and its expression is strongly correlated with the expression of the *PGDH1*^3^, which has been shown to be the major PGDH isoform in Arabidopsis (Benstein et al., 2013). This finding strongly suggest a role of *PSAT1* in the PPSB. As no T-DNA insertion lines are available for the *PSAT1* and *PSAT2* genes^4^, *PSAT1*-silenced lines (*ts-psat1.1* and *ts-psat1.2*) were generated for functional characterization using a microRNA-based approach (Felippes et al., 2012). Plants harboring an empty vector construct were used as control and further referred to EV.

**FIGURE 4:**
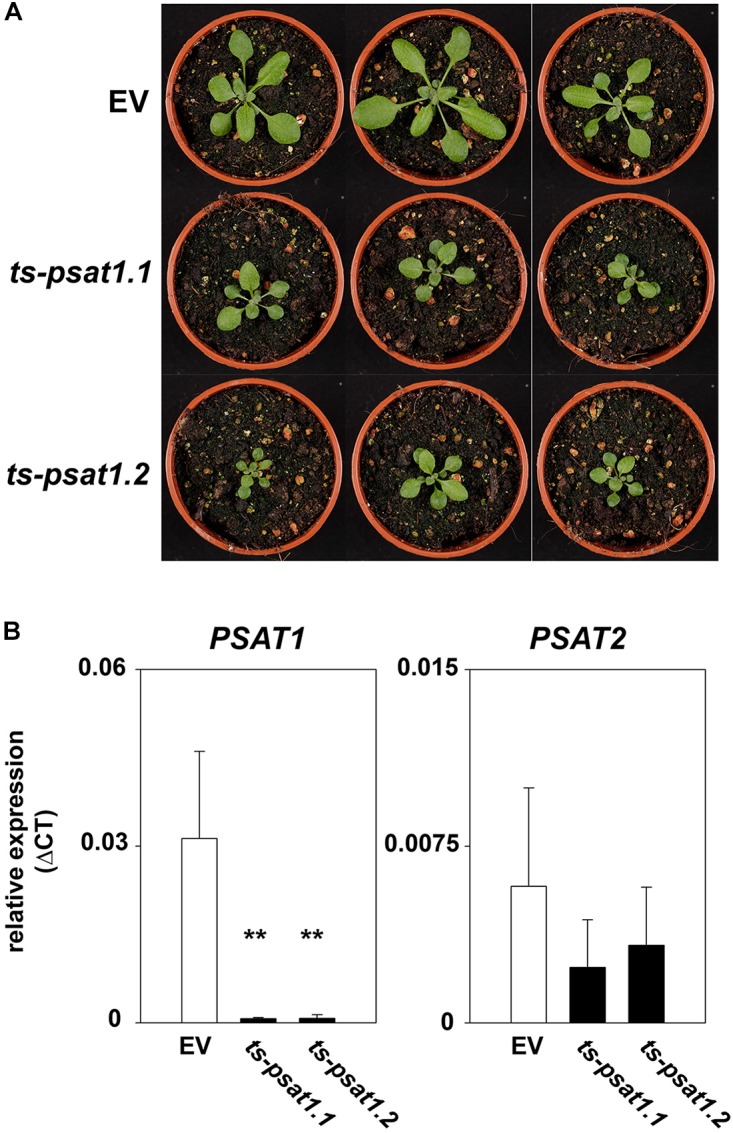
Phenotype of *PSAT1*-silenced plants. **(A)** Shoot phenotype of the two independent *PSAT1*-silenced plant lines *ts-psat1.1* and *ts-psat1.2* under standard growth conditions (day/night 16/8 h, 100 μmol_(photons)_ m^-2^ s^-1^) in comparison to the empty vector (EV) control plants. **(B)** Relative expression of *PSAT1* and *PSAT2* genes in *ts-psat1.1* and *ts-psat1.2* plants in comparison to EV control plants. The values represent the mean of four independent measurements. Error bars indicate the SD. ^∗∗^*P* ≤ 0.01, indicate significant differences between *PSAT1*-silenced lines and EV control.

**FIGURE 5:**
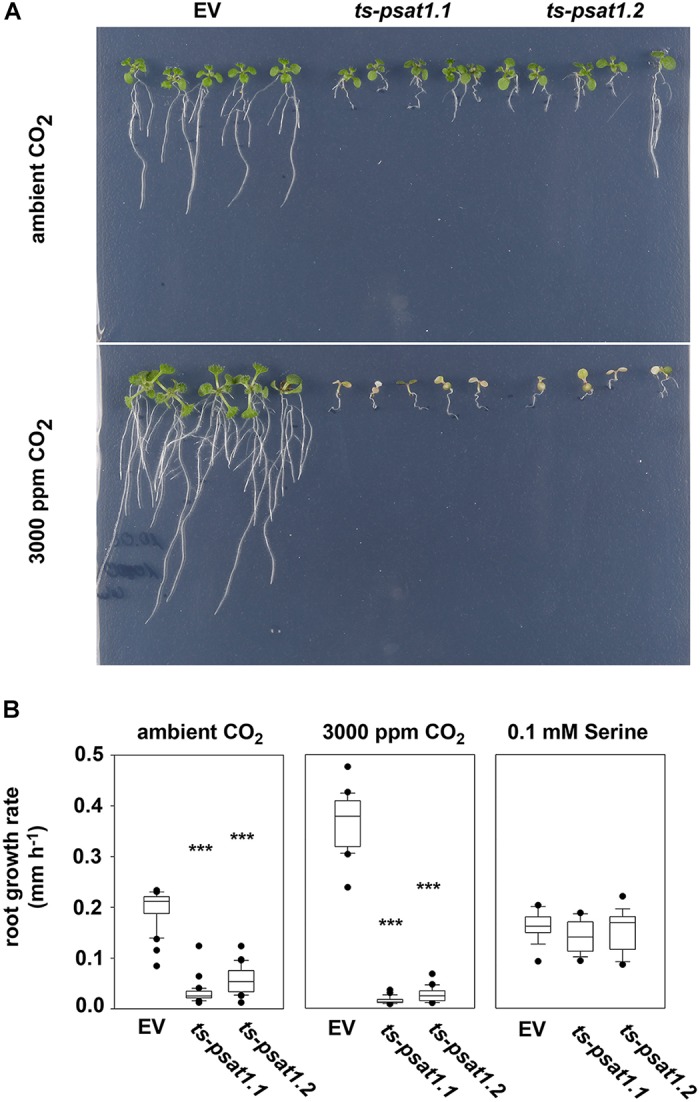
PSAT1 is essential for plant growth at elevated CO_2_. **(A)** Growth phenotype of *ts-psat1.1* and *ts-psat1.2* lines at ambient (380 ppm) and elevated (3000 ppm) CO_2_. **(B)** The root growth rate (mm h^-1^) of *ts-psat1.1* and *ts-psat1.2* plant lines in comparison with empty vector (EV) control lines is shown. The root growth rate of plants grown at ambient CO_2_ (left), elevated CO_2_ (middle) and in the presence of 0.1 mM serine (right) was determined daily. Therefore, 9 days old seedlings were transferred from ambient CO_2_ to elevated CO_2_ for 8 days. In parallel 6 days old seedlings were transferred from horizontal plates to vertical plates supplemented with 0.1 mM serine and the root growth rate was determined every day for 11 days. ^∗∗∗^*P* ≤ 0.001, indicate significant differences between *PSAT1*-silenced lines and EV control.

Overexpression of the artificial *PSAT1*-silencing construct in Arabidopsis leads to a significant reduction of *PSAT1* expression, which subsequently results in a strong inhibition of growth (Figures 4A,B). In contrast, the expression of the second *PSAT* gene was unaltered in *PSAT1*-silenced plants (Figure 4B). When these plants were grown at standard light conditions [100 μmol (photons) m^-2^ s^1^] the leaf area and therefore the rosette diameter was much smaller compared to the shoot of the EV control plants (Figure 4A). But not only growth of the aerial part of *PSAT1*-silenced lines was impaired, also the growth rate of the primary root was significantly reduced from 0.1977 mm h^-1^ for EV control plants to 0.0295 mm h^-1^ and 0.0539 mm h^-1^ for *ts-psat1.1 and ts-psat1.2* lines, respectively (Figures 5A,B).

To confirm that the growth phenotype observed for *PSAT1*-silenced plants is due to serine deficiency, plants were transferred from plates containing ½ MS medium to plates additionally supplemented with 0.1 mM serine, after germination. Determination of the primary root growth rate of plants grown on medium supplemented with serine revealed no significant difference between the EV control plants and *ts-psat1.1* and *ts-psat1.2* lines.

In addition, the primary root growth rate of plants transferred for 8 days from ambient to elevated CO_2_ was analyzed (Figures 5A,B). The primary root growth rate of EV control plants was significantly enhanced from 0.1977 mm h^-1^ at ambient CO_2_ to 0.3528 mm h^-1^ at elevated CO_2_ conditions. In contrast, the primary root growth rate of *ts-psat1.1* and *ts-psat1.2* lines was reduced from 0.0295 and 0.0539 to 0.0138 and 0.0261 mm h^-1^, respectively (Figure 5B). The primary root growth rate of *ts-psat1.1* and *ts-psat1.2* lines was determined at the first 2 days after transferred to elevated CO_2_ only, as later growth of both lines was arrested (Figures 5A,B). In general, *PSAT1*-silenced plants gradually stop growth of the shoot and the root after transfer to elevated CO_2_.

### Knockdown of *PSAT1* Alters Amino Acid Metabolism in Plants

The PSAT enzyme catalyzes the transfer of the amino group of glutamate to 3-phosphohydroxypyruvate, yielding in 2-oxoglutarate and 3-phosphoserine (Figure 1). Therefore, the phosphoserine pathway is intimately associated with glutamate metabolism in plants. To investigate the influence of PSAT1-deficiency on amino acid metabolism, the content of free amino acids was quantified in shoot and root tissue of control and *PSAT1*-silenced plants.

**FIGURE 6:**
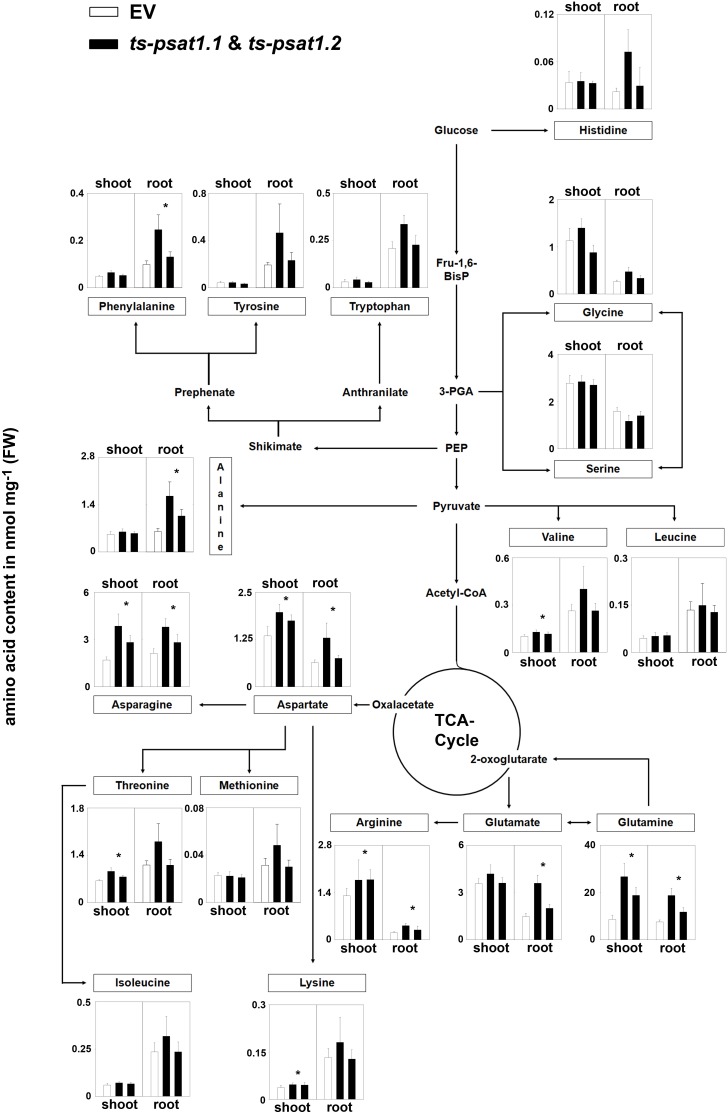
Metabolic analysis of *PSAT1*-silenced lines. The amino acid content in shoot and root tissue of *PSAT1*-silenced lines (*ts-psat1.1*, *ts-psat1.2*) and empty vector (EV) transformed control plants are shown. The values represent the mean of four independent biological replicates. Error bars indicate the SD. ^∗^*P* ≤ 0.05, indicate significant differences between *PSAT1*-silenced lines and EV control.

The quantification of amino acids revealed no significant changes in the content of serine and glycine in root and shoot tissue in *ts-psat1.1* and *ts-psat1.2* lines in comparison to control plants (Figure 6). Similarly, the content of histidine, tyrosine, tryptophan, leucine, isoleucine and methionine was also not altered in *PSAT1*-silenced plants. However, the content of some other amino acids, such as glutamine, arginine, aspartate, and asparagine was substantially increased in shoot and root tissue of these lines, whereas the content of lysine, threonine and valine was higher only in shoot tissue whereby the content of glutamate, glycine, alanine and phenylalanine was increased only in root tissue.

Altogether, these findings indicate that PSAT function is not only restricted to serine biosynthesis in plants.

### The PPSB Is Essential for Light and Sugar-Dependent Growth Promotion in *Arabidopsis thaliana*

**FIGURE 7:**
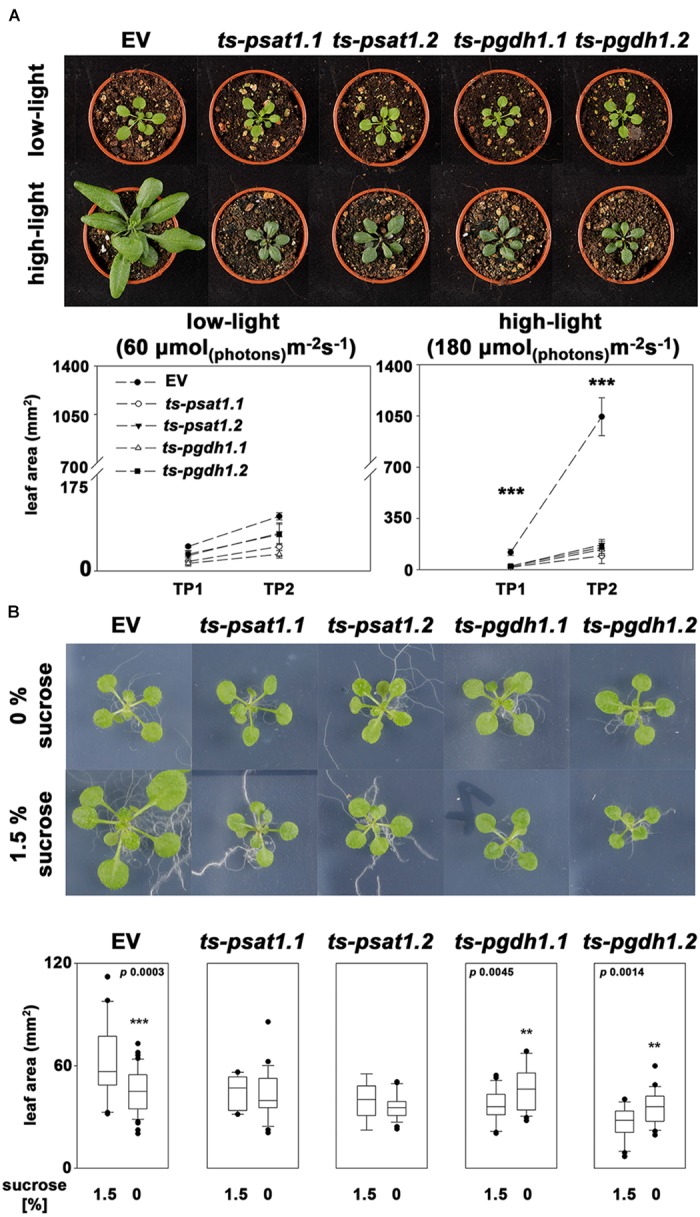
The PPSB is essential for light and sugar-dependent growth promotion. **(A)** The leaf area was determined of empty vector (EV), *PSAT1*-silenced (*ts-psat1.1*, *ts-psat1.2*) and *PGDH1*-silenced (*ts-pgdh1.1*, *ts-pgdh1.2*) plants grown at low (60 μmol _(photons)_ m^-2^ s^-1^) and high (180 μmol _(photons)_ m^-2^ s^-1^) light conditions. The leaf area was analyzed at two time points, 7 days after germination (TP1) and 14 days after germination (TP2). ^∗∗∗^*P* ≤ 0.001, significant differences between *PSAT1*- or *PGDH1*-silenced lines and the EV control. **(B)** The effect of externally applied sucrose on plant growth was studied in EV, *PSAT1*-silenced and *PGDH1*-silenced plants. Plants were grown on ½ MS medium with or without supplementation of 1.5% sucrose and growth promotion was determined by measuring the leaf area. ^∗∗∗^*P* ≤ 0.001, ^∗∗^*P* ≤ 0.01, and ^∗^*P* ≤ 0.05 indicate significant differences in leaf area between plants grown in the presence of 1.5% sucrose and plants grown on medium without sucrose.

Plant growth is intimately associated with the ability to convert light into biochemical energy to assimilate CO_2_ for the production of biomass. Increasing light intensity in a physiological range, results in enhanced biomass production as photosynthesis runs more efficiently. In addition, serine biosynthesis in plants is closely linked with photorespiration and therefore with photosynthesis. To test whether elevated photosynthesis can improve growth of PPSB-deficient plants, the leaf area of *PSAT1* and *PGDH1*-silenced lines grown at 60 μmol m^-2^ s^-1^ (low-light) and 180 μmol m^-2^ s^-1^ (high-light) was determined as a measure of growth analyzed and compared with the respective control plants (Figure 7A). The leaf area was determined of plants 7 and 14 days after germination.

**FIGURE 8:**
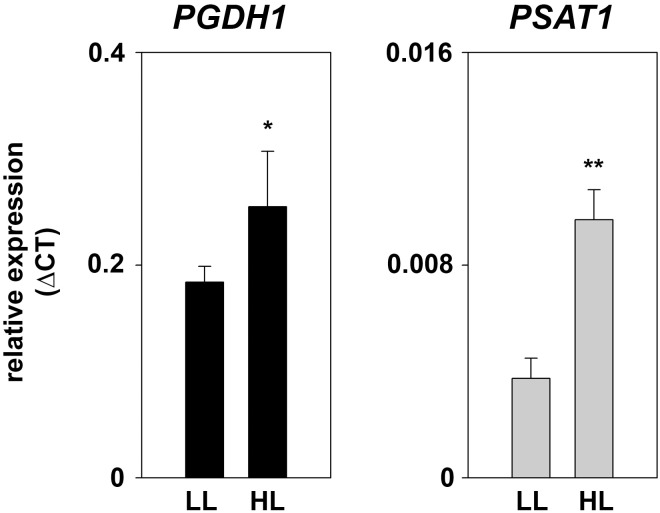
Expression of PPSB genes at different light conditions. Expression of *PGDH1* and *PSAT1* genes in plants grown at low-light (LL; 60 μmol _(photons)_ m^-2^ s^-1^) and high-light (HL; 180 μmol _(photons)_ m^-2^ s^-1^) conditions. Bars represent the mean of three biological replicates and error bars indicate SD. Asterisks designate significant differences (^∗^*P* ≤ 0.05; ^∗∗^*P* ≤ 0.01) between LL and HL treatment.

At low-light conditions growth of PPSB-deficient lines was slightly reduced compared to control plants, but the differences were not significant for all analyzed lines. The increase in growth after additional 7 days was generally low at low light conditions. However, 14 days after germination growth of PPSB-deficient lines was significantly lower than growth of the control plants. This effect was much more pronounced when the plants were cultivated at high-light conditions. Already at 7 days after germination all PPSB-deficient lines were significantly smaller than the control plants and this effect increased substantially after additional 7 days. To further investigate the importance of the PPSB for the light-dependent plant growth promotion, we analyzed the expression of PPSB genes under low and high-light conditions. The expression of *PGDH1* and *PSAT1* was significantly elevated in plants grown under high-light conditions (Figure 8). These results are supported by publicly available expression data, showing a positive correlation between light intensity and PPSB gene expression (Supplementary Figure 2).

Growth promotion due to increased light intensity is directly associated with elevated photosynthesis and the result of raised synthesis of carbohydrates and their enhanced availability as energy and carbon source for biomass production (Slattery et al., 2018). Therefore, we tested the effect of carbohydrate feeding on growth promotion of PPSB-deficient lines and control plants (Figure 7B). To avoid strong influences of the light conditions, the carbohydrate feeding experiments were conducted under low-light. Under these conditions, growth of control plants on plates supplemented with 1.5% sucrose was significantly higher than of plants grown without external sugar. In contrast, sucrose-induced growth promotion could not be observed in PPSB-deficient plants. The importance of the PPSB for sugar-dependent growth promotion is further supported by the induced expression of PPSB genes after transfer of the plant to sugar-containing plates (Supplementary Figure 3).

Altogether, our data indicate that the biosynthesis of serine by the phosphorylated pathway is rate limiting for plant growth.

### Amino Acids and Starch Accumulate Under High-Light Condition in Shoots of PPSB-Deficient Plants

**FIGURE 9:**
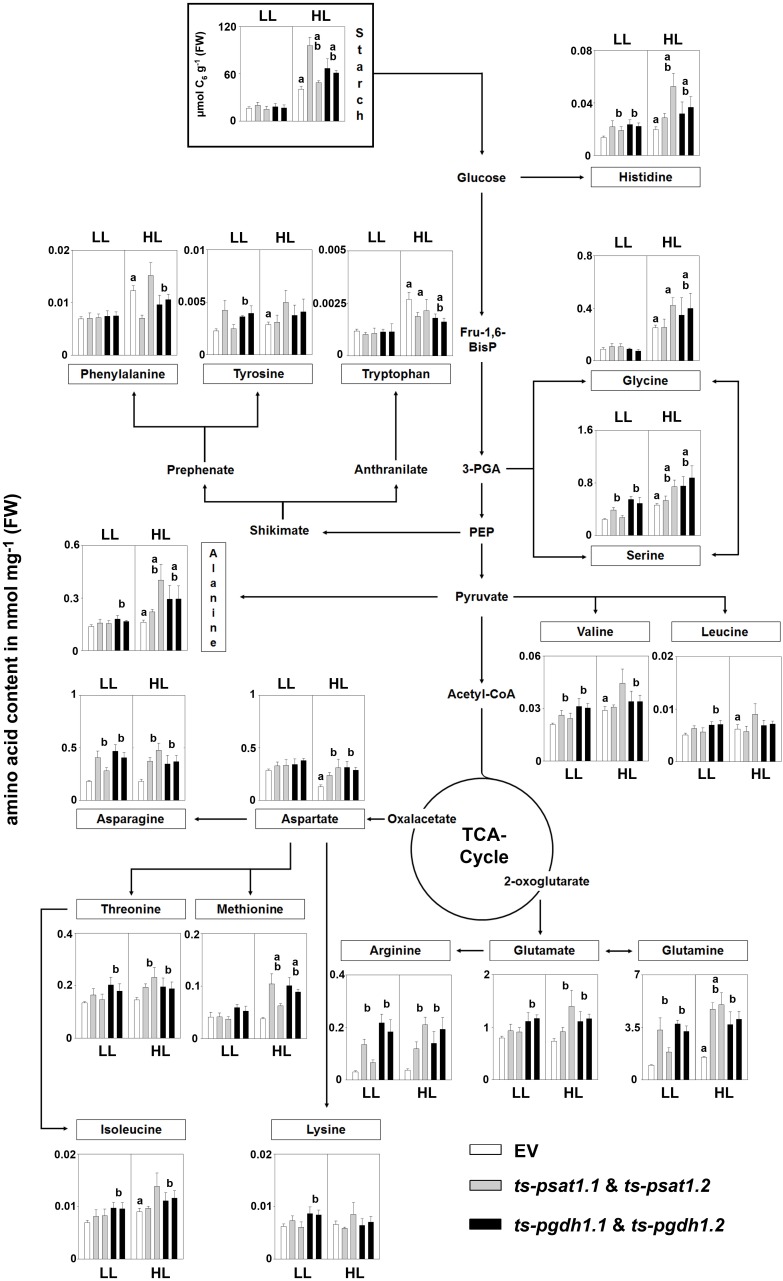
Metabolic analysis of *PSAT1* and *PGDH1*-silenced lines grown under low- and high-light conditions. The amino acid content in shoot tissue of *PSAT1*-silenced lines (*ts-psat1.1*, *ts-psat1.2*), *PGDH1*-silenced lines (*ts-pgdh1.1*, *ts-pgdh1.2*) and empty vector (EV) transformed control plants grown at low-light (60 μmol _(photons)_ m^-2^ s^-1^) or high-light (180 μmol _(photons)_ m^-2^ s^-1^) conditions are shown. The values represent the mean of four independent biological replicates. Error bars indicate the SD. a, indicate significant differences (*P* ≤ 0.05) between high-light and low-light grown plants and b, indicate significant differences (*P* ≤ 0.05) between *PSAT1* or *PGDH1*-silenced lines and the EV control plants.

The analysis of amino acid contents in plants showed a substantial increase in glycine, serine, histidine, alanine, valine, tryptophan, isoleucine, leucine, tyrosine, and glutamine in control plants grown under high-light conditions compared to plants grown under low-light conditions (Figure 9), whereas the contents of the other amino acids was not altered in these plants. In PPSB-deficient plants most of the amino acids, such as serine, histidine, valine, leucine, alanine, asparagine, threonine, isoleucine, lysine, glutamate, arginine, and glutamine accumulated stronger under low-light conditions than in control plants, but only for arginine, valine, asparagine, histidine, serine and glutamine the alteration was significant for both, *PGDH1* and *PSAT1*-silenced lines. In most cases, except for leucine and lysine, the amino acid content in PPSB-deficient plants became significantly higher than in control plants under high-light conditions only.

In plants grown under elevated light conditions the carbon assimilation and therefore also the synthesis of starch is enhanced (Schmitz et al., 2014). Starch is a major carbon and energy storage in plants and positively correlates with plant growth (Sulpice et al., 2009). The analysis of the starch content revealed no significant difference between control and PPSB-deficient plants when grown under low-light conditions (Figure 9). Plants grown under high-light conditions accumulated significantly more starch, compared to those grown under low-light. In addition, the starch content was significantly higher in PPSB-deficient lines in comparison to control plants.

## Discussion

### The Arabidopsis Genome Possesses Two Genes Encoding for PSAT Enzymes

The presence of PSAT activity in plants has been described previously by several studies (Larsson and Albertsson, 1979; Reynolds et al., 1988) and the gene encoding for the PSAT enzyme has been cloned from spinach and Arabidopsis (Saito et al., 1997; Ho et al., 1998). However, recent advances in genome sequencing revealed that two PSAT isoenzymes, namely PSAT1 (*At4g35630*) and PSAT2 (*At2g17630*) are encoded within the Arabidopsis genome^5^ and both enzymes have a high similarity with known PSAT enzymes of other species (Figure 2). Database search revealed that several plant species contain more than only one gene encoding for a PSAT enzyme (Supplementary Figure 1), indicating that the presence of multiple PSAT isoforms is common for plants.

The Arabidopsis PSAT isoforms, PSAT1 and PSAT2, contain an N-terminal sequence with a very high consensus score for a putative target peptide^6^, suggesting that both enzymes are functional within plastids. This is supported by the finding of Ho et al. (1998), who showed that the target peptide of PSAT1 is able to guide GFP into plastids of Arabidopsis plants. These findings are in line with the plastidial localization of all other PPSB genes (Ho et al., 1998, 1999a,b; Benstein et al., 2013; Cascales-Minana et al., 2013). Thus, the whole sequence of the PPSB, including the PSAT reaction, is plastid localized.

### PSAT1 Represents the Major PSAT Isoform in *Arabidopsis thaliana*

Expression analysis revealed that the *PSAT1* gene is much higher expressed in shoot and root tissue of Arabidopsis than the *PSAT2* gene (Figure 3). The expression of *PSAT1* is high in the vasculature of leaves, the shoot apical meristem, the vasculature of the root and the root apical meristem. This expression pattern is very similar to the expression of *PGDH1*, the main PGDH isoform (Benstein et al., 2013; Toujani et al., 2013), suggesting that PSAT1 could be the main PSAT enzyme in Arabidopsis.

To test whether PSAT1 plays an important role in Arabidopsis, *PSAT1*-silenced lines (*ts-psat1.1* and *ts-psat1.2*) were generated. In these plants, the expression of *PSAT1* is strongly reduced, whereas expression of *PSAT2* is not significantly altered (Figure 4). Therefore, the strong growth phenotype of the silenced lines (Figure 4) can be attributed to the specific down regulation of the *PSAT1* expression and the loss of PSAT1 activity cannot be compensated by PSAT2.

Previous studies on the function of the PPSB showed that *PGDH1* represents the major isoform as knockout mutants of this gene are embryo-lethal and silencing lines are retarded in growth (Benstein et al., 2013). In contrast, *PGDH2* knock out mutants are viable and showed no obvious phenotype (Benstein et al., 2013; Toujani et al., 2013). Therefore, it is very likely that PSAT1 represents the main PSAT enzyme in Arabidopsis, whereas the PSAT2 enzyme might have a subordinate function only.

The growth phenotype of *PSAT1-*silenced plants is enhanced when growing plants under non-photorespiratory elevated CO_2_ conditions and can be rescued by growing the plants on medium supplemented with serine (Figure 5). This phenotype is very similar to the appearance of plants with reduced PGDH1 and PSP activity (Benstein et al., 2013; Cascales-Minana et al., 2013). Increasing the CO_2_ concentration has been shown to be an efficient method to inhibit the photorespiration as under these conditions the RuBisCO enzymes prefers the carboxylation reaction (Eisenhut et al., 2013). Therefore, the higher sensitivity of *PGDH1* and also *PSAT1*-silenced plants against elevated CO_2_ can be explained by the simultaneous inhibition of the photorespiratory and PPSB-mediated serine biosynthesis.

The co-existence of several pathways for serine biosynthesis suggests, that one pathway alone is insufficient in fulfilling the demand for serine in plants. The enhanced expression of *PGDH1* (Benstein et al., 2013) as well as *PSAT1* (Figure 3) in heterotrophic and *inter alia* in meristematic tissue suggests a function of the PPSB in serine provision in cells, which are only insufficiently supplied with serine produced by photorespiration. In proliferating cells of the meristem, the rate of nucleic acid synthesis is high and consequently, also the demand for nucleotides and S-adenosylmethionine (Douce et al., 2001; Jabrin et al., 2003; Sahr et al., 2005). Thus, serine synthesized by the PPSB might be important to provide one-carbon units for the tetrahydrofolate metabolism and therefore for the synthesis of S-adenosylmethionine, purine and pyrimidine bases in these cells.

According to the metabolite analysis, the content of serine was unaltered in *PSAT1*-silenced plants (Figure 6), similarly, to previous observations for *PGDH1-*silenced lines or conditional *psp1* mutant plants (Benstein et al., 2013; Cascales-Minana et al., 2013). However, the growth phenotype of the silenced plants can be rescued by external application of serine, indicating that serine deficiency is the major issue these plants have. Therefore, the majority of serine measured in *PSAT1*-silenced plants is most likely synthesized via photorespiration, whereas serine synthesized by the PPSB might be restricted to few cells only and account for a comparatively small part of the total serine content. Due to the resulting small impact on the total serine pool size, serine quantification on the whole plant level appears to be an inappropriate measure for PPSB-dependent serine synthesis.

The most obvious metabolic phenotype of *PSAT1*-silenced plants is the strong accumulation of glutamine and some other amino acids in shoot and root tissue (Figure 6). This phenotype is very similar to the phenotype observed for *PGDH1*-silenced plants (Benstein et al., 2013) and might be the result of a disturbed ammonium assimilation in these plants. The PSAT reaction represents a putative link between PPSB activity and ammonium assimilation. The PSAT enzyme catalyzes the transfer of the amino group from glutamate to 3-phosphohydroxypyruvate, yielding in one molecule 3-phosphoserine and one molecule 2-oxoglutarate. The 2-oxoglutarate released by the PSAT activity can be directly recycled into glutamate by the GOGAT enzyme in plastids. Deficiency in the PSAT activity might disturb this recycling mechanism, causing 2-oxoglutarate deficiency in plastids and subsequent accumulation of glutamine. To prevent accruing of toxic ammonium, plants possess additional pathways for ammonium assimilation by synthesizing aspartate, asparagine, and arginine (Potel et al., 2009). The elevated level of these amino acids supports our hypothesis, although other reasons for this phenotype cannot be excluded.

### The PPSB Is Essential for Light and Sugar-Dependent Plant Growth Promotion

Plant growth and development is intimately associated with the quantity of light. Under optimal growth conditions, e.g., water and nutrient availability, elevated light increases CO_2_ assimilation and consequently biomass production (Slattery et al., 2018). Interestingly, this effect is strongly impaired in PPSB-deficient plants (Figure 7). While growth of the control plants under elevated light conditions was massively increased compared to low-light conditions, growth of *PGDH1* as well as *PSAT1*-silenced plants was not substantially improved by high-light. This effect cannot be attributed to a general serine deficiency in these plants, as under high-light conditions the total serine content was even higher in *PGDH1* and *PSAT1*-silenced lines than in the control plants (Figure 9). The higher serine content is most likely the consequence of an elevated photorespiration, which is supported by the fact that also the glycine content was markedly raised under these conditions. Photorespiration is tightly associated with ammonium assimilation in plants (Coschigano et al., 1998). Elevated flux through the photorespiratory pathway could also explain why the content of most other amino acids was increased in shoots of PPSB-deficient plants. However, the strong growth phenotype of the silenced lines under high-light conditions in turn leads to the question, why the higher serine content is insufficient for rescuing the phenotype of PPSB-deficient plants? These results can only be explained by considering the presence of independent pools of serine in plants, which are not efficiently connected to each other. The growth arrest of PPSB-deficient plants under non-photorespiratory elevated CO_2_ conditions clearly shows that both serine biosynthetic pathways are interconnected. However, it is very likely that serine transport out of mesophyll cells, where photorespiration takes place, into the cells of the meristems, where the PPSB is highly active, is limiting.

Similarly to the effect of elevated light, external application of sugar also promotes plant growth (Blasing et al., 2005; Eckstein et al., 2012). Cultivation of plants on medium supplemented with 1.5% sucrose substantially improved growth of control plants, but not of PPSB-deficient lines. These results are in line with the observation that PPSB-deficient plant accumulate significantly more starch under high-light conditions than control plants (Figure 9). Hence, PPSB-deficient plants are not able to convert the energy provided in form of carbohydrates into biomass. The importance of the PPSB for plant growth is further supported by the fact that the expression of *PGDH1* and *PSAT1* was significantly up-regulated by high-light and the application of carbohydrates (Figure 8 and Supplementary Figures 2, 3).

Altogether, our findings indicate that serine biosynthesis by the PPSB is rate limiting for plant growth.

## Materials and Methods

### Plant Material and Cultivation Conditions

*PSAT1*-silenced lines (*ts-psat1.1* and *ts-psat1.2*) were generated by fusion of a *PSAT1* genomic fragment to the target site of the trans-acting small interfering RNA miR173 (Felippes et al., 2012). For this, a cDNA fragment of was amplified using a modified oligonucleotide that included the miR173 target sequence. The PCR amplicon was cloned into the pENTR/D-TOPO (Invitrogen) vector and recombined into the pAM-PAT-GWPro35S vector (Jakoby et al., 2006). The generated construct was transformed into the Agrobacterium tumefaciens strain GV3101 pmp90RK and subsequently into Arabidopsis Col-0. Transformed plants were germinated on soil and transgenic lines were selected by spraying with glufosinate (BASTA).

*PGDH1*-silenced lines (*ts-pgdh1.1* and *ts-pgdh1.2*) and the respective empty vector (EV) control plants were previously described by Benstein et al. (2013).

Plants were either grown on half-strength Murashige and Skoog medium without sucrose or on soil. The plants were stratified at 4°C in darkness for 2.5 and 4 days, respectively, before transfer to a plant growth chamber [22°C, 16 h light, 100 μmol (photon) m^-2^ s^-1^].

### Root Growth Analyses

For determination of the primary root growth rate, seeds of silenced and control plants were germinated for 4 days after stratification on ½ MS plates before transfer to vertical plates for additional 10 days of culture. To test whether external serine can rescue the root growth phenotype of *PSAT1*-silenced plants, seeds were germinated on ½ MS medium supplemented with 100 μM serine and later transferred to vertical plates supplemented with serine. The root growth was tracked over the whole growth period. For root growth measurement, images were taken using a standard digital camera (Nikon) and the evaluation of the pictures was performed by using the ImageJ software^7^.

### Expression Analysis by qRT-PCR

For the analysis of gene transcripts, RNA was extracted from plant material by hot phenol extraction and subsequent LiCl precipitation. Extracted RNA samples were treated with a TURBO DNA-free kit (Ambion) and were subsequently used for cDNA synthesis via the Bioscript Reverse Transcription kit (Bioline). Quantification of gene expression was achieved by quantitative Real Time Polymerase Chain Reaction using the 7300 Real-Time PCR System and SYBR Green (Applied Biosystems). Ubiquitin and actin served as housekeeping genes.

### Histochemical Analysis of Promoter:GUS Construct Lines

Plants expressing the β-glucuronidase (*GUS*) reporter gene under control of the native *PSAT1* or *PSAT2* promoter (*ProPSAT1:GUS; ProPSAT2:GUS*, Benstein et al., 2013) were cultivated for 10 days on ½ MS plates. For histochemical staining of GUS activity, seedlings were vacuum infiltrated (Vacuubrand GmbH) five times for 1 min in staining solution [0.1 M NaH_4_PO_4_ pH 7.2, 10 mM EDTA, 0.5 mM K-ferrocyanide (K_4_Fe(CN)_6_), 5 mM K-ferricyanide (K_3_Fe(CN)_6_), 0.1% Triton X-100 and 20 mM X-Gluc/DMSO] and thereafter incubated at 37°C overnight. Subsequently, seedlings were destained with 70% EtOH at 60°C, again incubated in fresh 70% EtOH and were then viewed under the binocular microscope (Leica S84PO) for GUS activity.

### Extraction and Measurements of Metabolites

For quantification of amino acids, lyophylized plant material was extracted with 80% ETOH and the extract was diluted 1:10 with water. Twenty microliters of the diluted extract were injected into the HPLC before HPLC analysis. Amino acids were separated using a HyperClone 3u ODS (C18)120 150 × 4.6 mm column (Phenomenex) connected to the Dionex UltiMateTM 3000 system (Thermo Fisher Scientific). The HPLC system held a discontinuous flow gradient comprising solvent A (8.8 mM NaPO4, pH 7.5, and 0.2% (v/v) tetrahydrofuran) and increasing proportion of solvent B (18.7 mM NaPO4, pH 7.5, 32.7% (v/v) MeOH and 20.6 % (v/v) acetonitrile), at a flow rate of 0.8 mL/min. Quantification was performed by pre-column derivatization with ortho-phtalaldehyde (OPA; Grace Davison Discovery Science) and subsequently quantified by fluorescence detection of the obtained derivatives according to Lindroth and Mopper (1979).

Starch was isolated from lyophylized plant material as described by Lin et al. (1988) and the content was determined with a coupled enzymatic assay as described earlier by Stitt (1989) using a Spectrafluor Plus plate reader (TECAN, Austria) in the absorbance mode.

## Author Contributions

SW and SK conducted the research and wrote the manuscript. SK designed the research.

## Conflict of Interest Statement

The authors declare that the research was conducted in the absence of any commercial or financial relationships that could be construed as a potential conflict of interest.

## Footnotes

1 http://aramemnon.uni-koeln.de

2 https://phytozome.jgi.doe.gov/pz/portal.html

3 http://atted.jp/

4 http://signal.salk.edu/cgi-bin/tdnaexpress

5 https://www.arabidopsis.org/

6 http://aramemnon.uni-koeln.de
