# Supplementary material for: Phosphoserine Aminotransferase1 Is Part of the Phosphorylated Pathways for Serine Biosynthesis and Essential for Light and Sugar-Dependent Growth Promotion
Source: Front Plant Sci. 2018 Nov 20;9:1712. doi: 10.3389/fpls.2018.01712 (PMC6256069; doi:10.3389/fpls.2018.01712)
Supplement: Supplementary file 1 [file Data_Sheet_1.pdf]

## SUPPLEMENTAL FIGURES

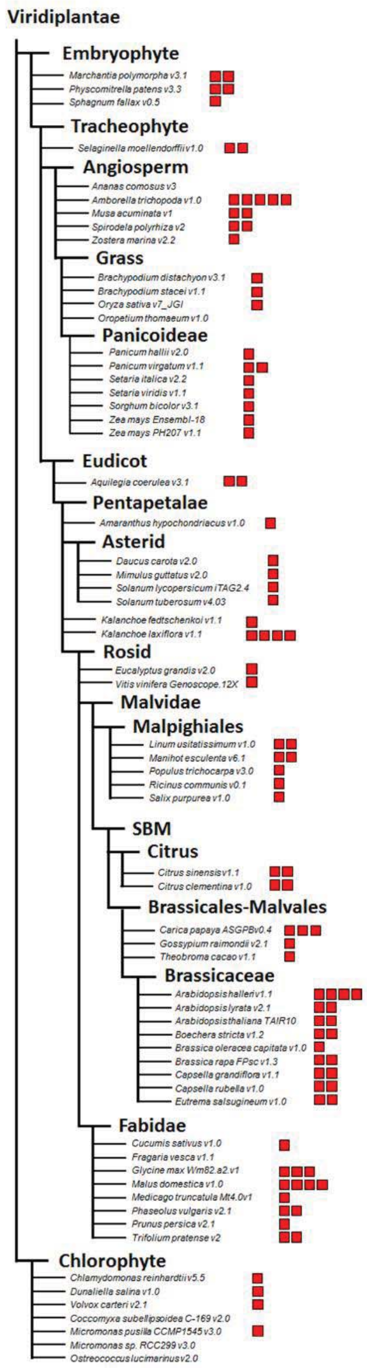


**Supplemental Figure 1: Presence of the *PSAT* genes in the clade *Viridiplantae***

The presence of the *PSAT* genes in the clade *Viridiplantae* is shown. Red boxes indicate the number of *PSAT* isoforms.

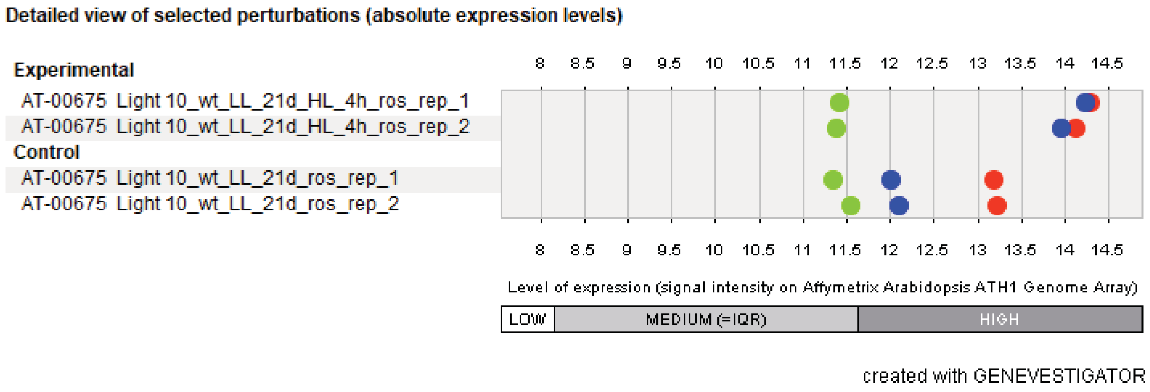


**Supplemental Figure 2: High-light-induced expression of PPSB genes**

Public available gene expression data are shown (https://genevestigator.com/gv/). The expression of *PGDH1* (red), *PSAT1* (blue) and *PSP* (green) is shown in shoot tissue of wild type plants grown for three weeks on soil under 16h low-light (30 µmol $_{(photons)}$ m$^{-2}$ s$^{-1}$) at 22°C / 8h dark at 18°C cycles, then exposed to high-light (16h (300 µmol $_{(photons)}$ m$^{-2}$ s$^{-1}$) at 22°C / 8h dark at 18°C) for 48h. The plants were shifted to high-light 3.5h after the beginning of the light period.

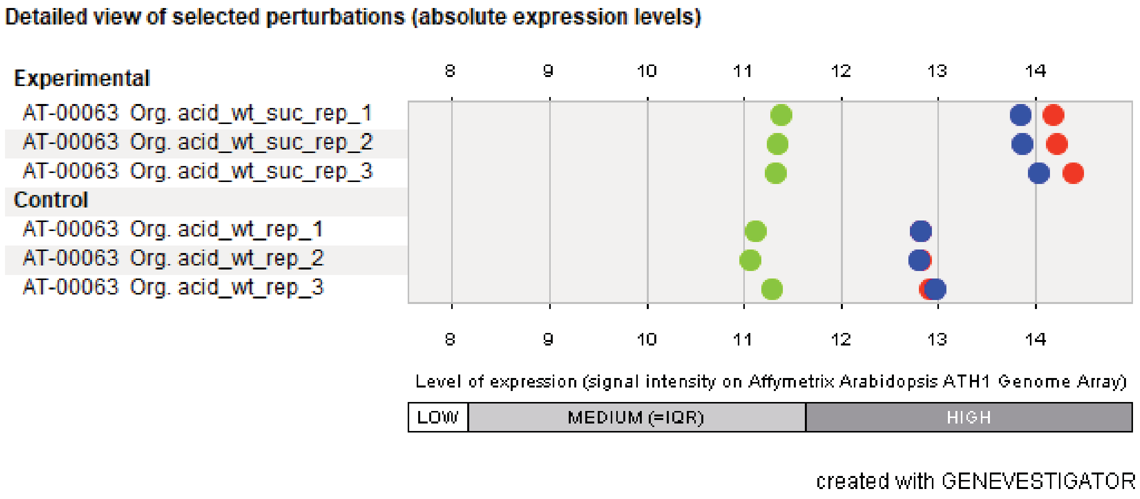


**Supplemental Figure 3: Sucrose-induced expression of PPSB genes**

Publicly available gene expression data are shown (https://genevestigator.com/gv/). The expression of *PGDH1* (red), *PSAT1* (blue) and *PSP* (green) is shown in seedlings of wild type plants grown on agar plates with (100 mM) or without exogenous sucrose for 3 days.
